# Body mass index trajectories among people with obesity and association with mortality: Evidence from a large Israeli database

**DOI:** 10.1002/osp4.475

**Published:** 2020-12-23

**Authors:** Orna Reges, Dror Dicker, Christiane L. Haase, Nick Finer, Tomas Karpati, Morton Leibowitz, Altynai Satylganova, Becca Feldman

**Affiliations:** ^1^ Clalit Research Institute Clalit Health Services Ramat Gan Israel; ^2^ Department of Preventive Medicine Feinberg School of Medicine Northwestern University Chicago Illinois USA; ^3^ Internal Medicine D Department and EASO Collaborating Center for Obesity Management Rabin Medical Center Hasharon Hospital Petach Tikva Israel; ^4^ Sackler School of Medicine Tel Aviv University Tel Aviv Israel; ^5^ Novo Nordisk A/S Søborg Denmark; ^6^ Holon Institute of Technology Holon Israel

**Keywords:** body mass index, databases, mortality, obesity, population studies, weight change

## Abstract

**Objective:**

Previous studies using longitudinal weight data to characterize obesity are based on populations of limited size and mostly include individuals of all body mass index (BMI) levels, without focusing on weight changes among people with obesity. This study aimed to identify BMI trajectories over 5 years in a large population with obesity, and to determine the trajectories' association with mortality.

**Methods:**

For inclusion, individuals aged 30–74 years at index date (1 January 2013) with continuous membership in Clalit Health Services from 2008 to 2012 were required to have ≥1 BMI measurement per year in ≥3 calendar years during this period, of which at least one was ≥30 kg/m^2^. Latent class analysis was used to generate BMI trajectories over 5 years (2008–2012). Cox proportional hazards models were used to assess the association between BMI trajectories and all‐cause mortality during follow‐up (2013–2017).

**Results:**

In total, 367,141 individuals met all inclusion criteria. Mean age was 57.2 years; 41% were men. The optimal model was a quadratic model with four classes of BMI clusters. Most individuals (90.0%) had stable high BMI over time. Individuals in this cluster had significantly lower mortality than individuals in the other trajectory clusters (*p* < 0.01), including clusters of people with dynamic weight trajectories.

**Conclusions:**

The results of the current study show that people with stable high weight had the lowest mortality of all four BMI trajectories identified. These findings help to expand the scientific understanding of the impact that weight trajectories have on health outcomes, while demonstrating the challenges of discerning the cumulative effects of obesity and weight change, and suggest that dynamic historical measures of BMI should be considered when assessing patients' future risk of obesity‐related morbidity and mortality, and when choosing a treatment strategy.

## 1 INTRODUCTION

The association between individuals' increasing high body mass index (BMI) and higher rates of clinical complications is well established.^1^ Various studies have shown an increase in mortality with increasing BMI^2^, ^3^, ^4^; furthermore, individuals with overweight or obesity are at greater risk of developing diseases such as type 2 diabetes (T2D)^5^ or cardiovascular (CV) disease^5^, ^6^, ^7^ than individuals with healthy weight. Consequently, a new international classification of disease (ICD) for obesity was recently proposed, based on a combination of pathophysiology, BMI, CV complications remediable by weight loss, and the severity of complications.^8^

Obesity‐related complications are also linked to increased mortality,^9^ and high direct and indirect healthcare costs.^10^, ^11^ According to international guidelines, BMI level and the presence of comorbidities are key criteria in treatment decisions for the management of obesity.^12^ However, much of the available evidence on obesity‐related complications is based on BMI measurements calculated at one time point, whereas the relationship between weight change over time and obesity‐related complications is less well characterized. Outside interventional clinical trials, studies examining how gaining, maintaining or losing weight differentially affects the risks for obesity‐related outcomes have yielded contradictory results. Several studies have shown an association between stable high or increasing weight trajectories and increased CV risk^13^ and mortality.^14^, ^15^ However, others have reported that fluctuating weight is linked to comparatively poorer outcomes in patients with T2D,^16^ and carries an overall greater CV risk^17^, ^18^ and higher mortality^18^ than stable weight.

In clinical trials and observational studies, weight loss is typically studied in the context of a specific medical^19^, ^20^ or surgical^21^, ^22^ intervention for the management of obesity over a certain time frame. Other approaches are required to provide information on weight change in a general population with obesity. However, accurate identification of weight trajectories based on real‐world data can be limited by both the breadth and the depth of patient data available for longitudinal analysis. Several previous studies have assessed longitudinal weight trajectories in adults and their association with selected health outcomes, but these have also been subject to some of the common limitations of observational studies. For example, many included relatively small populations,^14^, ^23^, ^24^, ^25^, ^26^, ^27^ selected individuals within a narrow age range,^14^, ^25^ or defined BMI trajectories for the general population rather than specifically for individuals with obesity.^14^, ^23^, ^24^, ^25^, ^26^, ^27^, ^28^

In the absence of extensive evidence, assessment of longitudinal BMI data in real‐world populations with obesity is highly valuable, both contributing to a more accurate clinical description of obesity phenotypes and strengthening the understanding of the impact of weight changes over time. The main objective of this study was to identify BMI trajectories over a 5‐year period in a large population with obesity, using a comprehensive Israeli electronic health record database, and to assess the association of BMI trajectories with all‐cause mortality.

## 2 METHODS

### 2.1 Data source

This study used historical data from Clalit Health Services,^29^ Israel's largest healthcare provider, serving approximately 54% of the population. Since 2002, Clalit Health Services has maintained a comprehensive, integrated, centralized electronic health records data warehouse, which covers 4.4 million members at the time of writing. Height and weight measurements have been included as quality measures in this database since 2008; therefore, weight measurements are routinely collected by healthcare practitioners. Mortality data from the Israeli Ministry of the Interior is integrated into the Clalit Health Services data warehouse.

### 2.2 Study design and population

**FIGURE 1:**
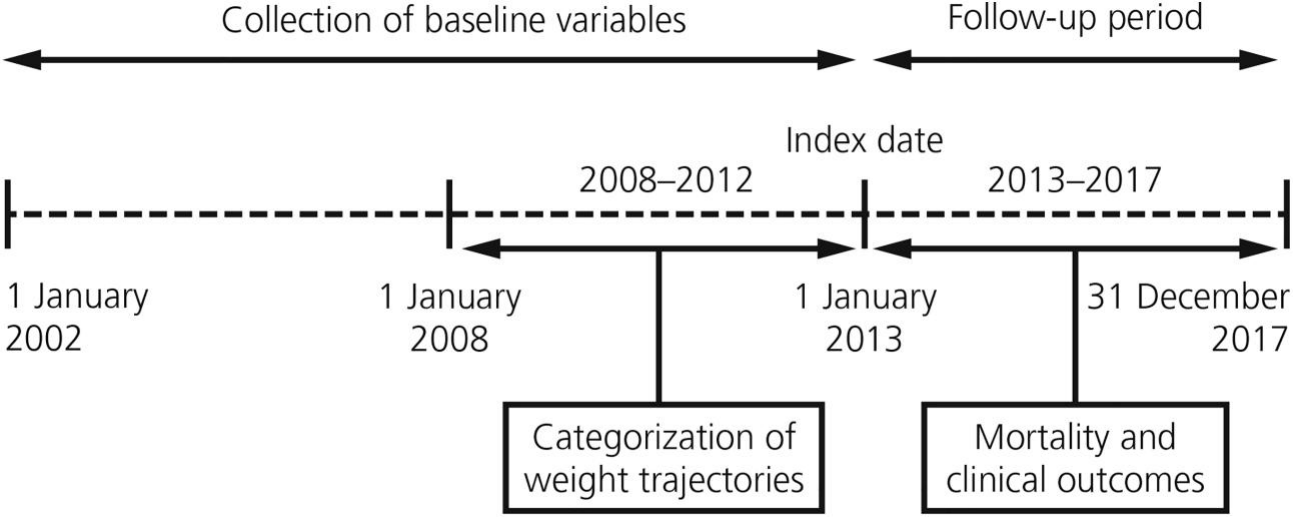
Study design

This was a retrospective cohort study, with the following dates and study periods (Figure 1):

1. baseline variable collection, 1 January 2002 to 31 December 2012 (when multiple instances of relevant data were available, those closest to index date were taken as baseline);
2. categorization of BMI trajectories and collection of weight‐loss intervention data, 1 January 2008 to 31 December 2012;
3. index date, 1 January 2013;
4. follow‐up period for outcomes, 1 January 2013 to 31 December 2017.

For inclusion, individuals aged 30–74 years at index date required continuous membership in Clalit Health Services during the 5‐years BMI trajectories categorization period. Individuals also required data allowing the calculation of at least one BMI measurement per year in a minimum of three calendar years during this period, of which at least one had to be ≥ 30 kg/m^2^. The 5‐years time frame of the categorization period allowed documentation of the minimum required number of weight measurements and was considered appropriate for determining weight changes. Individuals with a major amputation as of the index date were excluded from the study.

### 2.3 Baseline variables at index date

Baseline characteristics of interest included demographic characteristics and prevalence of obesity‐related comorbidities. A full list of baseline characteristics of interest can be found in the Supporting Information. Comorbidities were identified by ICD‐9 codes^30^ and free‐text descriptions. To examine whether medication use differed depending on weight trajectory, and whether weight trajectory was linked to specific interventions, purchase of certain pharmaceutical treatments (CV medication, blood‐glucose lowering drugs, and antidepressants) and weight‐loss interventions (medication, surgical intervention, and visit to a dietician) during the 5 years pre‐index date were also recorded.

### 2.4 Outcomes

The primary outcome was all‐cause mortality (yes/no only; cause not defined) during the 5 years post‐index date. Current and complete information regarding mortality events was obtained from Israel's Ministry of the Interior, which includes the entire Israeli population. Secondary outcomes included incidence of new diagnosis of T2D (defined using a Clalit Research Institute‐reported algorithm^31^), major adverse cardiac events (MACE; defined as incidence of myocardial infarction, unstable angina pectoris, any percutaneous transluminal coronary angioplasty or any coronary artery bypass graft) and chronic kidney disease.

### 2.5 Generation of trajectories

BMI measurements during 2008–2012 were calculated based on weight and height measurements in that time frame. Weight measurements recorded during pregnancy and up to 6 months afterward were excluded. Outliers from the BMI measurements data set were identified for removal by multivariate outlier analysis (R package mvoutlier version 2.0.8)^32^ and conditional decision tree analysis, defining the following exclusion rules: BMI >60 kg/m^2^, BMI <13 kg/m^2^, weight >175 kg, weight <33 kg, height ≤1.30 m, or height >2.10 m.

Latent class analysis (LCA), a tool commonly used for identifying and grouping a set of underlying subpopulations according to patterns based on the difference in longitudinal trajectories,^33^ was used to generate clusters of BMI trajectories for individuals in the study population. LCA was applied using R version 3.4.3^34^ and the latent class mixed models (lcmm) library version 1.7.8.^35^ The first BMI measurement in each of the five calendar years (of a minimum of three calendar years) was used to determine the BMI trajectory for each individual. To establish the suitability of this measure, the mean first BMI documented for each year was assessed for concordance with the other BMI‐related variables (first documented, last documented, maximum documented, minimum documented, and mean of all documented BMI). Both linear and quadratic LCA models were generated, with 2–6 clusters for each method; the model with the lowest Akaike information criterion (AIC) and Bayesian information criterion (BIC) was considered optimal.

### 2.6 Statistical analysis

The main characteristics of the total study population were described using proportions for categorical variables and means with standard deviation (SD) for continuous variables. To assess the unadjusted and adjusted associations between BMI (last measure and identified trajectories) and all‐cause mortality, a fixed Cox regression, with baseline (last) BMI and the BMI trajectory cluster as the main exposure, was used, with adjustment for potential confounders in the baseline variable collection period, including age; sex; immigration status; ethnicity; socioeconomic status; place of residence; marital status; and comorbidities at index date (see Figure 4 for complete list). Termination of follow‐up was defined as death or end of follow‐up period (31 December 2017). Individuals with known active cancer during 2008–2012 were excluded from this analysis. The assumption of proportional hazards was tested and a *p* value <0.05 with two‐sided test was used as the statistically significant threshold.

### 2.7 Ethical approval and use of data

This study using secondary data was approved by Clalit Health Services' institutional review board, in accordance with the Declaration of Helsinki. Data were used in accordance with the terms agreed to upon their receipt.

## 3 RESULTS

### 3.1 Study population and baseline characteristics

**FIGURE 2:**
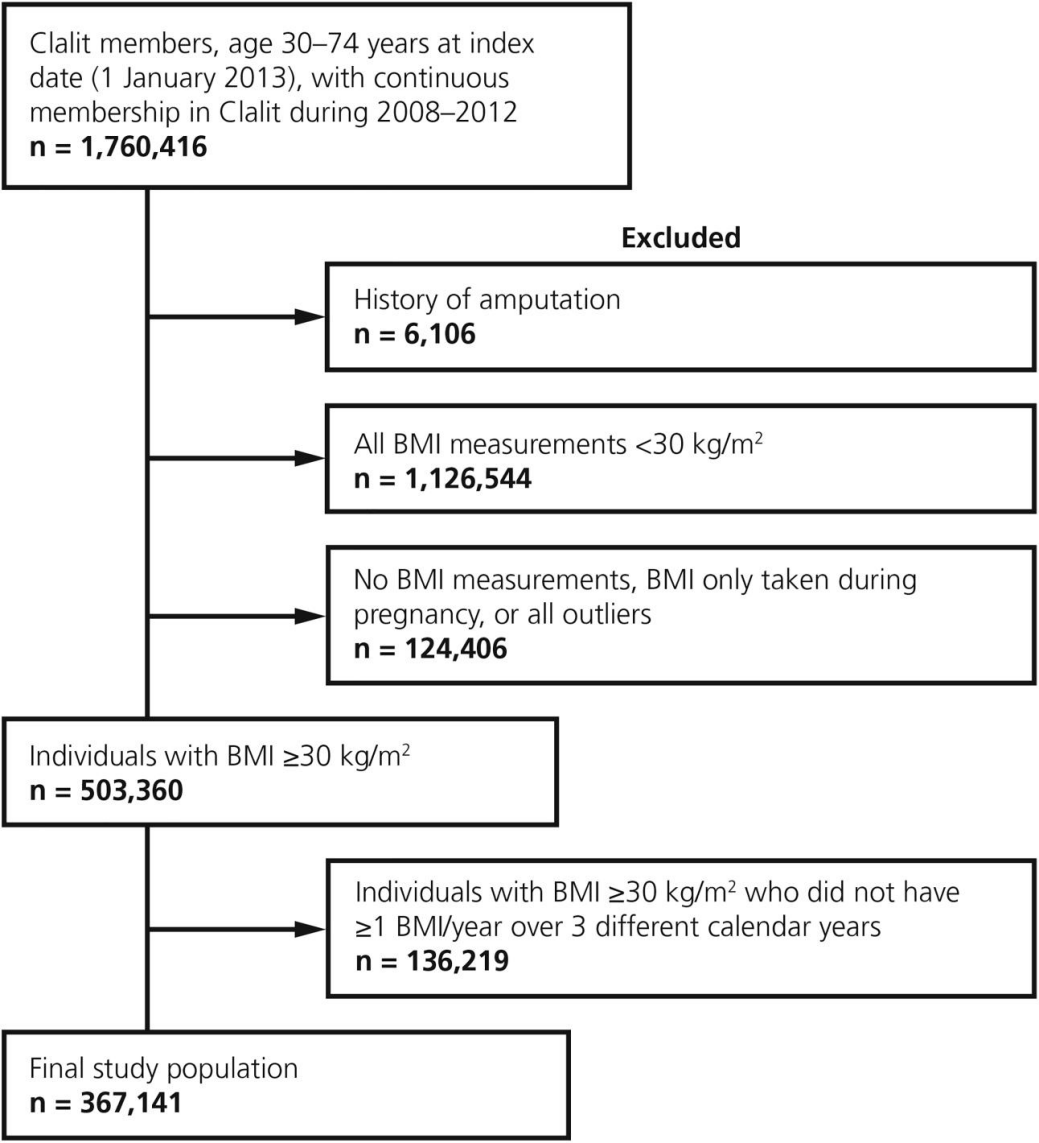
Patient flow. BMI, body mass index; Clalit, Clalit Health Services

A total of 1,760,416 individuals aged 30–74 years at index date with continuous membership in Clalit Health Services during the study period were identified in the database (Figure 2). Of these, 503,360 individuals met all the inclusion criteria and had at least one valid BMI measurement. However, 136,219 individuals did not have the required number of BMI values for trajectory analysis and were excluded. Consequently, the final study population consisted of 367,141 individuals; this corresponds to 73% of Clalit Health Services members with a BMI ≥30 kg/m^2^ (without a history of amputation or BMI measurements during pregnancy) (Figure 2).

Key baseline characteristics of the study population are summarized in Table 1. The mean age was 57.2 years (SD 10.8), the majority were women (59.0%) and mean Charlson comorbidity score was 1.5 (SD 1.7). Compared with those excluded from the study owing to missing BMI measurements, the study population was significantly older (57.2 vs. 44.2 years; *p* < 0.001) and had more comorbidities (Charlson score: 1.53 vs. 0.47; *p* < 0.001), and a greater proportion were women (59.0% vs. 52.1%; *p* < 0.001; Table 2).

**TABLE 1:** Baseline characteristics of the study population by BMI trajectory clusters

BMI trajectory cluster	Stable high	Very high, slightly increasing	Dynamic increasing–decreasing	Dynamic decreasing–increasing	Total
*n*	330,558	28,907	4889	2787	367,141
Baseline characteristics					
Age (years), mean (SD)	57.5 (10.7)	56.1 (10.8)	51.8 (12.1)	52.9 (12.3)	57.2 (10.8)
Women, *n* (%)	189,580 (57.4)	22,186 (76.7)	3087 (63.1)	1687 (60.5)	216,540 (59.0)
Charlson comorbidity score, mean (SD)	1.51 (1.7)	1.84 (1.9)	1.38 (1.8)	1.20 (1.6)	1.53 (1.7)
Sociodemographic characteristics					
Born in Israel, *n* (%)	196,575 (59.5)	18,363 (63.5)	3477 (71.1)	1949 (69.9)	220,364 (60.0)
Arab, *n* (%)	79,161 (23.9)	9209 (31.9)	1210 (24.7)	763 (27.4)	90,343 (24.6)
Low socioeconomic status, *n* (%)	146,310 (44.3)	15,389 (53.2)	2166 (44.3)	1316 (47.2)	165,181 (45.0)
Married, *n* (%)	209,649 (63.4)	15,691 (54.3)	2851 (58.3)	1677 (60.2)	229,868 (62.6)
Concurrent comorbidities of interest, *n* (%)					
Dyslipidemia	244,314 (73.9)	20,914 (72.3)	2999 (61.3)	1588 (57.0)	269,815 (73.5)
Hypertension	185,190 (56.0)	19,979 (69.1)	2322 (47.5)	1149 (41.2)	208,640 (56.8)
T2D	121,627 (36.8)	14,261 (49.3)	1602 (32.8)	687 (24.7)	138,177 (37.6)
Respiratory disorders	60,215 (18.2)	8028 (27.8)	1097 (22.4)	512 (18.4)	69,852 (19.0)
Ischemic heart disease	59,419 (18.0)	5005 (17.3)	611 (12.5)	329 (11.8)	65,364 (17.8)
Osteoarthritis	48,685 (14.7)	5851 (20.2)	521 (10.7)	258 (9.3)	55,315 (15.1)

Abbreviations: BMI, body mass index; SD, standard deviation; T2D, type 2 diabetes.

**TABLE 2:** Baseline characteristics of individuals with BMI ≥30 kg/m^2^ who were included in the study and those who were excluded owing to missing BMI records or absence of valid BMI measurements

	Included in this study	Individuals with BMI ≥30 kg/m^2^ without ≥1 BMI measurement/year over 3 different calendar years (excluded)	Individuals with no BMI measurement over the trajectory period (excluded)	*p* value
*n*	367,141	136,219	124,406	
Age (years), mean (SD)	57.24 (10.79)	44.19 (9.49)	40.72 (9.80)	<0.001^a^ ^,^ ^b^
Women, *n* (%)	216,540 (59.0%)	70,958 (52.1%)	67,091 (53.9%)	<0.001^a^ ^,^ ^c^
Socioeconomic status				
Low, *n* (%)	165,181 (45.0%)	65,279 (47.9%)	44,236 (35.6%)	<0.001^a^ ^,^ ^c^
Medium, *n* (%)	137,492 (37.4%)	47,700 (35.0%)	49,748 (40.0%)	
High, *n* (%)	63,818 (17.4%)	22,996 (16.9%)	29,581 (23.8%)	
Unknown, *n* (%)	650 (0.2%)	244 (0.2%)	841 (0.7%)	
Charlson comorbidity score, mean (SD)	1.53 (1.74)	0.47 (0.86)	0.23 (0.62)	<0.001^a^ ^,^ ^b^

Abbreviations: BMI, body mass index; SD, standard deviation.

^a^ All comparisons are statistically significant.

^b^ Based on one‐way analysis of variance test. Bonferroni tests were carried out as a post hoc analysis.

^c^ Based on chi‐squared test.

### 3.2 BMI measurements

A total of 2,846,323 valid BMI measurements were recorded for the study population over the 5‐years trajectory categorization period, with a mean number of measurements per person of 7.75 (SD 5.76) for the entire 5 years (Table 3). The mean first BMI measurement was similar to the mean of all other BMI measurements taken (∼33 kg/m^2^ across all other BMI‐related variables), demonstrating its robustness for use in this analysis.

**TABLE 3:** Description of valid recorded BMI measurements during 2008–2012 among the study population (*n* = 367,141)

Year valid BMI recorded	2008	2009	2010	2011	2012	Total
*n* (%)	520,308 (18.3)	557,163 (19.6)	578,217 (20.3)	591,222 (20.8)	599,413 (21.1)	2,846,323 (100.0)
Number of measurements per person, mean (SD)	1.42 (1.63)	1.52 (1.70)	1.57 (1.74)	1.61 (1.77)	1.63 (1.84)	7.75 (5.76)
Individuals with ≥1 valid BMI, *n* (%)	289,072 (78.7)	304,727 (83.0)	311,081 (84.7)	316,190 (86.1)	318,433 (86.7)	367,141 (100.0)

Abbreviations: BMI, body mass index; SD, standard deviation.

### 3.3 BMI trajectories

A total of 10 different LCA models (five linear and five quadratic models) were used to examine the performance of 2–6 clusters for each type. The optimal model, with AIC 7,695,024.91 and BIC 7,695,232.11, was a quadratic model with four distinct clusters of BMI trajectories **(**Table 4).

**TABLE 4:** Respective performance outputs (Akaike information criterion and Bayesian information criterion) by model type and number of classes

Model type	Number of classes	AIC	BIC
Linear	2	7,813,456.27	7,813,543.51
	3	7,750,428.69	7,750,548.65
	4	7,717,196.48	7,717,349.15
	5	7,701,355.10	7,701,540.50
	6	7,712,062.24	7,712,280.35
Quadratic	2	7,806,350.11	7,806,470.07
	3	7,740,709.00	7,740,872.59
	**4**	**7,695,024.91**	**7,695,232.11**
	5	7,729,536.73	7,729,787.56
	6	7,721,797.61	7,722,092.06

*Note:* The model resulting in the lowest AIC and BIC is shown bold.

Abbreviations: AIC, Akaike information criterion; BIC, Bayesian information criterion.

**FIGURE 3:**
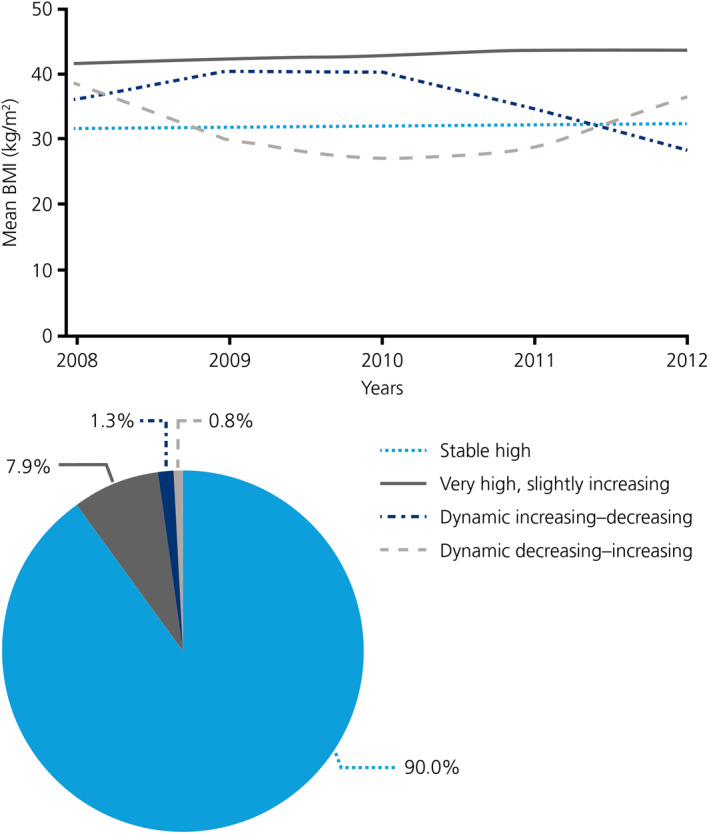
Longitudinal BMI trajectory clusters over the 5‐years study period and proportions of individuals in each cluster. BMI, body mass index

Most individuals with obesity at baseline displayed stable high BMI over time (330,558; 90.0% of the study population). A further 28,907 individuals (7.9%) had very high, slightly increasing BMI over the study period. Very few individuals had dynamic increasing–decreasing BMI (4889; 1.3%) and an even smaller proportion had dynamic decreasing–increasing BMI (2787; 0.8%) over time (Figure 3).

### 3.4 Baseline characteristics across BMI trajectories

Baseline characteristics across BMI trajectories are shown in Table 1. In general, individuals with dynamic BMI trajectories were younger (51.8 years in the increasing–decreasing and 52.9 years in the decreasing–increasing BMI trajectory clusters) than those with stable high (57.5 years) or very high, slightly increasing (56.1 years) BMI trajectories. More individuals with a very high, slightly increasing BMI trajectory were women compared with those with a stable high BMI trajectory, a dynamic increasing–decreasing BMI trajectory and a dynamic decreasing–increasing BMI trajectory (76.6% vs. 57.4%, 63.1% and 60.5%, respectively). A higher percentage of individuals in the very high, slightly increasing BMI trajectory cluster had a baseline history of hypertension, T2D, respiratory disorders and osteoarthritis, compared with individuals in the other BMI trajectory clusters; the only exception was dyslipidemia, which had a slightly higher prevalence in the stable high trajectory cluster. Overall, those with dynamic BMIs generally had lower rates of comorbidities compared with individuals who had stable high BMI over time. The mean Charlson comorbidity score was highest among those with a very high, slightly increasing BMI trajectory (1.8 [SD: 1.9]) and lowest in the group with a dynamic decreasing–increasing BMI trajectory (1.2 [1.6]).

### 3.5 Weight‐loss interventions in the 5 years pre‐index date

Table 5 shows pharmaceutical treatments and weight‐loss interventions received by individuals in each trajectory cluster in the 5 years pre‐index date. In this study population, 4.4% had undergone bariatric surgery in the five years pre‐index date, with pronounced differences between trajectories: the highest percentage was found in the dynamic increasing–decreasing BMI trajectory cluster (30.3%), followed by the very high, slightly increasing BMI trajectory cluster (22.9%), dynamic decreasing–increasing BMI trajectory cluster (12.5%), and stable high BMI trajectory cluster (2.3%).

**TABLE 5:** Weight‐loss interventions and pharmaceutical treatments in the 5 years pre‐index date, by BMI trajectory clusters

BMI cluster	Stable high	Very high, slightly increasing	Dynamic increasing–decreasing	Dynamic decreasing–increasing	Total
*n*	330,558	28,907	4889	2787	367,141
Weight‐loss interventions					
Bariatric surgery, *n* (%)	7681 (2.3)	6622 (22.9)	1483 (30.3)	347 (12.5)	16,133 (4.4)
Weight‐loss medication, *n* (%)	8694 (2.6)	1518 (5.3)	321 (6.6)	84 (3.0)	10,617 (2.9)
Visit to dietitian					
Number of individuals with ≥1 visit, *n* (%)	126,312 (38.2)	14,269 (49.4)	2379 (48.7)	867 (31.1)	143,827 (39.2)
Number of visits, mean (SD)	5.44 (7.22)	6.15 (7.8)	6.28 (7.39)	4.7 (8.15)	5.52 (7.3)
Pharmaceutical treatment					
Cardiovascular system, *n* (%)					
Beta‐blocking agents	84,772 (25.6)	9209 (31.9)	776 (15.9)	430 (15.4)	95,187 (25.9)
Calcium channel blockers	56,281 (17.0)	6368 (22.0)	508 (10.4)	260 (9.3)	63,417 (17.3)
Agents acting on the renin‐angiotensin system	132,141 (40.0)	14,512 (50.2)	1196 (24.5)	670 (24.0)	148,519 (40.5)
Lipid‐modifying agents	166,334 (50.3)	14,595 (50.5)	1424 (29.1)	877 (31.5)	183,230 (49.9)
Injectable blood‐glucose lowering drugs, *n* (%)					
Insulins	21,435 (6.5)	2859 (9.9)	167 (3.4)	107 (3.8)	24,568 (6.7)
Non‐insulins	5397 (1.6)	1071 (3.7)	36 (0.7)	17 (0.6)	6521 (1.8)
Blood‐glucose lowering drugs, excluding insulins, *n* (%)					
Any blood‐glucose lowering drug	83,468 (25.3)	9935 (34.4)	691 (14.1)	373 (13.4)	94,467 (25.7)
Biguanides	70,763 (21.4)	8670 (30.0)	608 (12.4)	327 (11.7)	80,368 (21.9)
Sulphonamides	20,415 (6.2)	2570 (8.9)	117 (2.4)	80 (2.9)	23,182 (6.3)
Combinations of oral blood‐glucose lowering drugs	13,064 (4.0)	1195 (4.1)	69 (1.4)	34 (1.2)	14,362 (3.9)
Thiazolidinediones	24 (0.0)	8 (0.0)	0 (0.0)	0 (0.0)	32 (0.0)
Dipeptidyl peptidase‐4 inhibitors	4474 (1.4)	423 (1.5)	19 (0.4)	10 (0.4)	4926 (1.3)
Antidepressants, *n* (%)	34,200 (10.3)	3515 (12.2)	513 (10.5)	276 (9.9)	38,504 (10.5)

Abbreviations: BMI, body mass index; SD, standard deviation.

In total, 2.9% of individuals had a record of purchasing weight‐loss medications; the highest rates were found in the dynamic increasing–decreasing (6.6%) and very high, slightly increasing (5.3%) BMI trajectories. More than 30% of individuals in each BMI trajectory had visited a dietitian during the same time frame. Individuals in the very high, slightly increasing BMI trajectory cluster used pharmaceutical treatments such as blood‐glucose lowering interventions, CV disease medication and antidepressants more frequently than individuals in the other trajectory clusters; individuals in both dynamic trajectory clusters reported considerably less frequent use of these.

### 3.6 BMI trajectories and all‐cause mortality during the follow‐up period

**FIGURE 4:**
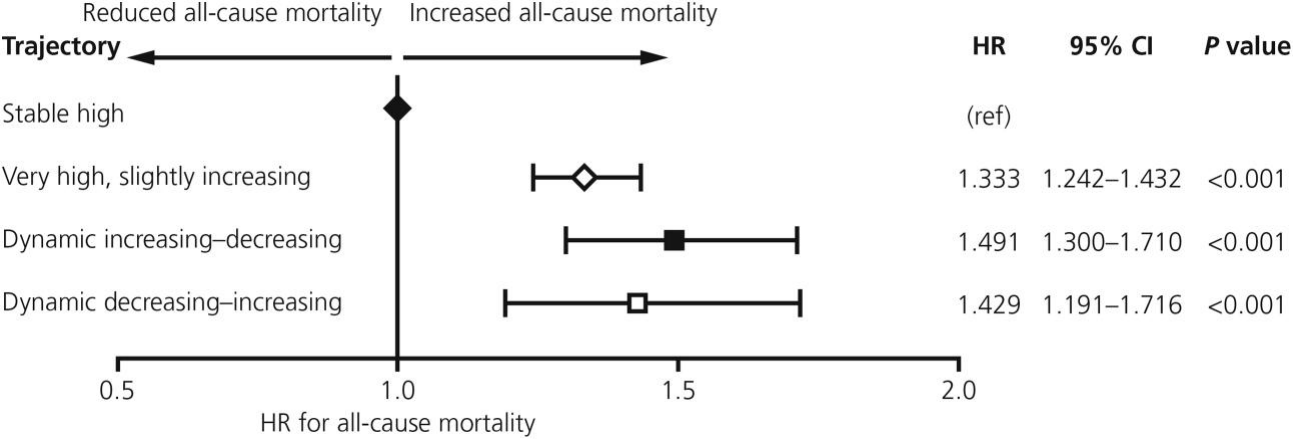
Association between BMI trajectory clusters and all‐cause mortality among individuals with obesity (fixed Cox regression). Dependent variable: all‐cause mortality during the years 2013–2017; independent variables: BMI trajectory clusters. Covariates at index date: baseline BMI; age; sex; immigration status; ethnicity; socioeconomic status; place of residence; marital status and comorbidities at index date (active cancer, ischemic heart disease, stroke, chronic heart failure, pulmonary embolism, atrial fibrillation, hypertension, time with diabetes, hypothyroidism, chronic obstructive pulmonary disease, urinary incontinence, chronic kidney disease, osteoarthritis, Charlson score [without age], glucose, low‐density lipoprotein‐cholesterol levels, high‐density lipoprotein‐cholesterol levels, and blood pressure). BMI, body mass index; CI, confidence interval; HR, hazard ratio

The highest incidence of all‐cause mortality during the follow‐up period was observed among individuals in the very high, slightly increasing BMI trajectory cluster (7.2%), followed by 6.3% in the dynamic increasing–decreasing BMI trajectory cluster, 5.2% in the dynamic decreasing–increasing BMI trajectory cluster and 4.6% in the stable high BMI trajectory cluster (Table 6). A multivariable Cox proportional hazards model confirmed an independent association between BMI trajectories and all‐cause mortality, above and beyond baseline BMI measurements, with a 1.33‐fold risk (hazard ratio [HR], 95% confidence interval [CI] 1.24–1.43) in individuals with a very high, slightly increasing BMI trajectory, 1.49‐fold risk [1.30–1.71] among individuals with a dynamic increasing–decreasing BMI trajectory, and a 1.43‐fold risk [1.19–1.72] among individuals with a dynamic decreasing–increasing BMI trajectory, compared with individuals with a stable high BMI trajectory (*p* < 0.01 for all associations; Figure 4).

**TABLE 6:** Incidence of clinical outcomes by BMI trajectory cluster during the follow‐up period (2013–2017)^a^

BMI trajectory	Stable high	Very high, slightly increasing	Dynamic increasing–decreasing	Dynamic decreasing–increasing	Total
All‐cause mortality					
Mortality, *n* (%)	15,166 (4.6)	2084 (7.2)	309 (6.3)	145 (5.2)	17,704 (4.8)
Mortality, *n* (%), excluding individuals with active cancer	12,144 (3.9)	1790 (6.5)	213 (4.6)	117 (4.4)	14,264 (4.1)
Mortality/1000 person‐years (95% CI)	9.4 (9.2–9.5)	14.9 (14.3–15.6)	13.1 (11.7–14.6)	10.7 (9.0–12.5)	9.9 (9.7–10.0)
T2D					
Individuals without T2D at index date	208,931	14,646	3287	2100	228,964
Incidence of T2D, *n* (%)	25,361 (12.1)	2611 (17.8)	157 (4.8)	182 (8.7)	28,311 (12.4)
MACE					
Individuals without IHD at index date, *n*	271,139	23,902	4278	2458	301,777
Incidence of MACE, *n* (%)	8909 (3.3)	625 (2.6)	80 (1.9)	75 (3.1)	9689 (3.2)
CKD					
Individuals without CKD at index date, *n*	311,422	26,671	4700	2657	345,450
Incidence of CKD, *n* (%)	18,669 (6.9)	2016 (7.6)	213 (5.0)	127 (4.8)	21,025 (7.0)
Incidence of RTT, *n* (%)	248 (0.1)	24 (0.1)	4 (0.1)	2 (0.1)	278 (0.1)

^a^ Numbers reported in this table have not been adjusted for potential confounding differences between groups.

Abbreviations: BMI, body mass index; CI, confidence interval; CKD, chronic kidney disease; IHD, ischemic heart disease; MACE; major adverse cardiac events; RTT, renal replacement therapy; T2D, type 2 diabetes.

The unadjusted and adjusted significant associations between baseline BMI and all‐cause mortality (HR [95% CI]: 1.03 [1.02–1.03]; *p* < 0.001, and 1.01 [1.01–1.02]; *p* < 0.01, respectively) disappeared when the BMI trajectories were introduced as an independent variable into the model (HR 1.00 [1.00–1.01]; *p* = 0.342).

### 3.7 Secondary outcomes

During the follow‐up period there was a higher incidence of T2D among individuals in the very high, slightly increasing BMI trajectory cluster (17.8%), compared with 12.1% in the stable high BMI trajectory cluster, 8.7% in the dynamic decreasing–increasing BMI trajectory cluster, and 4.8% in the dynamic increasing–decreasing BMI trajectory cluster. The incidences of MACE and chronic kidney disease by BMI trajectory cluster during the follow‐up period are shown in Table 6.

## 4 DISCUSSION

This retrospective cohort study utilized population‐based electronic health record data from 367,141 adults with obesity. Four BMI trajectories over a 5‐year period were generated using LCA, and the stable high weight trajectory, consisting of 90% of individuals, was found to be associated with lower mortality compared with very high, slightly increasing or dynamic BMI trajectories. The finding that most individuals with obesity maintained a stable high BMI supports previous studies conducted in the general population, which have described weight loss in fewer than 5% of individuals, whereas 70%–90% maintained a stable weight.^25^, ^26^

These findings are also in accord with studies indicating an association between weight stability and lower mortality.^25^, ^36^, ^37^ This could be because fewer individuals with stable high BMI experienced severe obesity (BMI >40 kg/m^2^), compared with those in other trajectory clusters. Alternatively, it may reflect differences between trajectories in terms of total cumulative time with obesity and the prevalence of comorbidities. The higher risk of all‐cause mortality among individuals with dynamic BMI may also result from BMI changes triggered by an underlying disease, which is suggested by the small proportion of patients receiving therapeutic weight‐loss treatment in this study, because unintentional weight loss often indicates that a patient may have a serious, hitherto undiagnosed, condition.

Evidence on how weight loss affects morbidity and mortality is mixed. Several studies have reported that weight loss and accompanying weight fluctuations can lead to increased morbidity^16^, ^17^, ^18^; however, in a UK epidemiological study, individuals who had lost and regained weight had a lower CV risk than those with stable obesity, overweight or normal weight, suggesting that weight loss, even if not sustained, could result in long‐term CV benefit.^38^ Similarly, intentional weight loss in clinical trials is often associated with decreased mortality,^19^ but a link between weight loss and increased mortality has been previously reported in an observational study.^39^ Overall, these results demonstrate the challenges of discerning the cumulative effects of obesity and weight change on morbidity and mortality in clinical practice, particularly when very few individuals with obesity receive weight‐loss treatment or lose weight.

Whereas previous studies have been conducted in relatively small cohorts based on general populations, this study included over 350,000 adults with obesity. The Clalit Health Services database is a large and comprehensive data source that has been used in previous observational studies^40^, ^41^, ^42^ showing that approximately 27% of members have obesity.^11^ This is similar to reported obesity rates of approximately 23% across Europe^43^; therefore, this study population was broadly representative of Western obesity rates. Another strength of this analysis was the length of the study period. Although the majority of individuals maintained stable weight, the 5‐years time frame used for categorization of BMI trajectories was sufficient to detect meaningful weight change in a subset of the population.

Despite the use of a robust data source, this study had some limitations. Firstly, obtaining sufficient BMI measurements to allow resolution of multiple different weight trajectories is an inherent challenge of using real‐world data for longitudinal studies. A possible approach to increase the number of BMI measurements to identify further patterns of weight change would be the inclusion of individuals with overweight as well as obesity. The capture of weight data is also vulnerable to bias: data from younger individuals with fewer underlying conditions, who have less contact with primary care, may not be captured on a regular basis. In this study, the disparities in baseline characteristics between the included population and those lacking the required number of BMI measurements indicate that some selection bias was indeed present. Another limitation of this study is intrinsic to LCA. Although widely used to identify homogeneous clusters within heterogeneous populations, LCA is an unsupervised method that does not allow model refinement based on outcomes. The differences among the clusters identified in this study indicate that the algorithm was able to capture relevant patterns; however, the different sizes of these clusters made it difficult to assign clinical meaning or establish whether weight loss was intentional or unintentional.

It would be useful to examine the subgroups in each cluster in this data set in greater depth, to discern between populations receiving therapeutic weight‐loss interventions and those with unintentional weight loss related to chronic diseases. This would also allow for characterization of subpopulations who did not derive clinical benefit from, or did not receive, interventions. To further understand the clinical implications of the association between BMI trajectories and all‐cause mortality, the study methodology could be repeated in a population that intentionally tried to lose weight. In addition, individuals who maintain a healthy weight over time could be included as a reference group. The BMI trajectory could also be included as a time‐dependent variable in future models, to account for changes in BMI during the follow‐up period. By conducting such analyses, the underlying causes for both the observed BMI trajectories and the associated patterns in mortality could be more fully elucidated.

Taken together, these results provide useful insights for clinicians, policy‐makers, and researchers, and contribute to our understanding of weight change dynamics at a population level and their association with outcomes. The large percentage of individuals who maintained a stable high weight may indicate that most people with obesity are either not accessing any interventions, or the ones that they access are ineffective, demonstrating the need to develop more effective weight‐loss strategies. Finally, the results suggest that dynamic historical measures of BMI should be taken into account in clinical risk stratification when assessing patients' future risk of obesity‐related morbidity and mortality.

## CONFLICT OF INTEREST

Christiane Lundegaard Haase, Nick Finer and Altynai Satylganova are employees of Novo Nordisk A/S, and Nick Finer and Altynai Satylganova are shareholders of Novo Nordisk A/S. Morton Leibowitz is an employee of Clalit Research Institute. Orna Reges was an employee of Clalit Research Institute at the time of the analysis and is now a post‐doctoral research fellow at the Northwestern University, Chicago. Tomas Karpati was an employee of Clalit Research Institute at the time of the analysis and is now an employee of the Holon Institute of Technology. Dror Dicker is an employee of Hasharon Hospital, Petach Tikva, and Tel Aviv University. Becca Feldman was an employee of Clalit Research Institute at the time the analyses were conducted.

## Supporting information

Supplementary Material

Supplementary Material
