# Supplementary material for: Body mass index trajectories among people with obesity and association with mortality: Evidence from a large Israeli database
Source: Obes Sci Pract. 2020 Dec 23;7(2):148–58. doi: 10.1002/osp4.475 (PMC8019279; doi:10.1002/osp4.475)
Supplement: Supplementary file 1 — Supplementary Material [file OSP4-7-148-s001.docx]

**Body Mass Index Trajectories among People with Obesity and Association with Mortality: Evidence from a Large Israeli Database**

**Authors**

Orna Reges^1,2^, Dror Dicker^3,4^, Christiane Lundegaard Haase^5^, Nick Finer^5^, Tomas Karpati^1,6^, Morton Leibowitz^1^, Altynai Satylganova^5^, and Becca Feldman^1^

**Author affiliations**

^1^ Clalit Research Institute, Clalit Health Services, Ramat Gan, Israel

^2^ current address: Department of Preventive Medicine, Feinberg School of Medicine, Northwestern University, Chicago, IL, USA

^3^ Internal Medicine D Department and EASO Collaborating Center for Obesity Management, Hasharon Hospital, Rabin Medical Center, Petach Tikva, Israel

^4^ Sackler School of Medicine, Tel Aviv University, Tel Aviv, Israel

^5^ Novo Nordisk A/S, Søborg, Denmark

^6^ current address: Holon Institute of Technology, Holon, Israel

**Supporting information**

**Supporting methods**

*Demographic characteristics, comorbidities, pharmaceutical treatments and weight-loss interventions data collected during the baseline period*

Socio-demographics:

- - age
  - sex
  - immigration status
  - ethnicity
  - socioeconomic status
  - residence
  - marital status.

Comorbidities:

- - cardiovascular disease (myocardial infarction; unstable angina; stable angina; coronary artery bypass graft; angioplasty; ischemic heart disease; ischemic stroke; congestive heart failure; pulmonary embolism; peripheral artery disease; atrial fibrillation; hypertension)
  - metabolic disorders (glycemic status; dyslipidemia; hyperthyroidism; hypothyroidism)
  - respiratory disorders (asthma; chronic obstructive pulmonary disease [COPD]; obstructive sleep apnea [OSA])
  - urinary system disorders (urinary incontinence and chronic kidney disease [CKD])
  - musculoskeletal disorders (osteoporosis; osteoarthritis)
  - laboratory markers of chronic diseases and health behavior (HbA_1c_ concentration; plasma glucose level; blood lipid levels; thyroid function tests; blood pressure; smoking status)
  - Charlson comorbidity index.

Pharmaceutical treatments:

- - cardiovascular medication
  - blood-glucose lowering medications
  - antidepressants.

Weight reduction interventions:

- - surgical intervention
  - weight-loss medications
  - visit to a dietitian.
